# Chiral Metasurface Multifocal Lens in the Terahertz Band Based on Deep Learning

**DOI:** 10.3390/mi14101925

**Published:** 2023-10-13

**Authors:** Jingjing Wang, Sixue Chen, Yihang Qiu, Xiaoying Chen, Jian Shen, Chaoyang Li

**Affiliations:** 1 School of Electronic Science and Technology, Hainan University, Haikou 570228, China; 21220854000174@hainanu.edu.cn (J.W.); sixuechen@hainanu.edu.cn (S.C.); yihangqiu@hainanu.edu.cn (Y.Q.); 21220854000136@hainanu.edu.cn (X.C.); 2 State Key Laboratory of Marine Resource Utilization in South China Sea, Hainan University, Haikou 570228, China

**Keywords:** chiral metasurface, circular dichroism, deep learning, monofocal lens, multifocal lens

## Abstract

Chiral metasurfaces have garnered significant interest as an emerging field of metamaterials, primarily due to their exceptional capability to manipulate phase distributions at interfaces. However, the on-demand design of chiral metasurface structures remains a challenging task. To address this challenge, this paper introduces a deep learning-based network model for rapid calculation of chiral metasurface structure parameters. The network achieves a mean absolute error (MAE) of 0.025 and enables the design of chiral metasurface structures with a circular dichroism (CD) of 0.41 at a frequency of 1.169 THz. By changing the phase of the chiral metasurface, it is possible to produce not only a monofocal lens but also a multifocal lens. Well-designed chiral metasurface lenses allow us to control the number and position of focal points of the light field. This chiral metasurface, designed using deep learning, demonstrates great multifocal focus characteristics and holds great potential for a wide range of applications in sensing and holography.

## 1. Introduction

Terahertz waves are between microwaves and infrared waves, with stronger penetration than infrared waves, higher imaging resolution than microwaves, and safer than X-rays. They cover the frequency range of 0.1~10 THz and the corresponding wavelength range of 30~3000 μm. The special position of terahertz waves in the electromagnetic spectrum gives them the advantages of low photon energy, rich spectral information, and wide bandwidth. These features have resulted in terahertz waves being widely used in nondestructive flaw detection [1], safety inspection [2], medical imaging [3], and other fields. However, the size of the optics is a challenging issue in these applications. With developments in the integration and miniaturization of optical systems, it is difficult for conventional optical devices to be ultra-thin and ultra-light due to the principle of phase modulation and material limitations. Fortunately, the emergence of chiral metasurfaces provides a new solution to this problem.

Chiral metasurfaces are novel two-dimensional artificial materials consisting of unit structures with subwavelength dimensions, also known as left-handed materials [4,5,6,7]. They can be efficiently electromagnetically controlled by tuning the dielectric constant to produce great chirality [8,9]. Chirality is prevalent in nature and has the property of mirror asymmetry. In the field of optical research, the interaction of polarized light with matter produces two special optical phenomena: CD [10,11,12] and optical activity (OA) [13,14,15,16]. OA can change the direction of light polarization and is mainly used to detect the purity of living organisms.CD, which mainly manifests itself as the different absorption of left- and right-circularly polarized light, has a wide range of applications in fields such as holography [17,18], focusing, and imaging [19].

Initial monofocal designs were based on chiral metasurfaces with metallic structures, but their efficiency was usually low due to the inherent losses of metals. In recent years, researchers have developed an all-silicon chiral metasurface that is well protected from metal losses and has high amplitude transmittance, which enables efficient single-focusing designs. To date, some progress has also been made in multifocal focus designs. One approach is to calculate the phase distribution of a single point source on the metasurface according to the lens phase distribution formula and arrange multiple monofocal lenses to realize the potential of the multifocal lens [20]. However, this device is bulky and complicated to design. Chen et al. [21] arranged nanorod antennae into a metasurface to realize multipoint focusing in the near-infrared band, but the diffraction efficiency was low (about 2%). He et al. [22] used Y-G calculation to obtain the phase distribution on the metasurface and achieved good multipoint focusing results. Both of the above-mentioned focusing lens designs are based on phase recovery [23], where the optimal solution for the phase distribution of multiple point sources on the metasurface is obtained through multiple iterative calculations on the metasurface and the focusing surface. This method is computationally intensive and sometimes tends to fall into local optimal solutions [21,22]. The design of metasurface phase distributions for multifocal focusing using computational holography is not only less computationally intensive; the iterative approach is also more advantageous compared to the more complex case of multifocal distributions.

With the rapid development of artificial intelligence (AI), deep learning [24,25,26], as an important part of AI, has been widely used in computer vision [27,28], feature extraction [29,30], natural language processing [31,32], and other fields. Meanwhile, many deep learning methods have also been applied to perform structure-assisted design on metasurfaces [33,34,35,36,37]. Bowen Zheng et al. [38] used a deep neural network (DNN) approach that could predict the possible amplitude and phase coverage of the considered metasurface. The physical insights gained from the proposed DNN can be used to achieve a more efficient and accurate metasurface design. Parinaz Naseri et al. [39] used the machine learning (ML) method to solve the one-to-many mapping problem and automated the inverse design of two- and three-layer metasurfaces. Using this approach, optimization problems with single or multiple constraints can be solved in cases where interlayer coupling is non-negligible and synthesis is cumbersome with traditional methods. Yingshi Chen et al. [40] utilized a deep learning method of adaptive batch normalization (BN) layers to achieve the reverse design of a graphene 2D metasurface.

This paper presents a deep learning-assisted design method for on-demand design of the structural parameters of chiral metasurfaces. This method avoids the process of scanning large amounts of data in traditional design and also reduces the time of data acquisition. A small quantity of data obtained from simulations are utilized to train the network so that the network is capable of inverse designing chiral metasurface. In the inverse design of the trained network, only the desired maximum CD is designed, and the network can directly output multiple sets of structural parameters that satisfy the target CD. In addition, the method can obtain arbitrary structural parameters under a specific structure, thus filling in CDs that may be missed during parameter scanning more efficiently. Furthermore, we have designed multiple chiral metasurfaces with efficient CDs via this method. This work provides a new concept for the design of chiral metasurfaces.

## 2. Theory

### 2.1. Monofocal Lens

For an arbitrary chiral metasurface, its transmission properties can be expressed using the Jones matrix as follows:

(1) $$T=\left(\begin{array}{cc} t_{xx} & t_{xy}\\ t_{yx} & t_{yy} \end{array}\right)$$

where $t_{ij}$ denotes the propagation coefficient in the *i*-direction of the linear polarization incident to the *j*-direction. We define a matrix of rotation as R(θ):

(2) $$R\left(\theta \right)=\left(\begin{array}{cc} cos\theta & -sin\theta \\ sin\theta & cos\theta \end{array}\right)\;$$

where θ is the angle of rotation. When RCP $E_{in}=E_{0}·{\left[\begin{array}{cc} 1 & 0 \end{array}\right]}^{T}$ is vertically incident, $t_{xy}=t_{yx}=0$, and the following equation is obtained:

(3) $$E_{out}=\frac{1}{2}\left(t_{xx}+t_{yy}\right)\left(\begin{array}{c} 1\\ 0 \end{array}\right)+\frac{1}{2}(t_{xx}-t_{yy})e^{-i2\theta }\left(\begin{array}{c} 0\\ 1 \end{array}\right)$$

It can be seen that there are two components: the first term is the isotropic circularly polarized light after the transmission of LCP at the chiral metasurface; and the second term is the crossed circularly polarized light with a phase change of 2θ phases. The principle is the same for left circularly polarized light in metasurface transmission parameters and right circularly polarized light.

From the above principle, it can be seen that phase control can be achieved by changing the rotation angle of the base atom, which is also called PB phase control [41,42,43]. By controlling the phase of each base atom, focusing can be achieved. In this case, the phase mutation for focusing should satisfy the following:

(4) $$\theta =-\frac{2\pi }{\lambda }(\sqrt{x^{2}+y^{2}+f^{2}}-f)$$

where λ is the operating wavelength of 250 µm, the focal length of f is 4800 µm, and θ is the variation of the phase. In the study of chiral metasurfaces, the phase can be manipulated independently to control the intensity of electromagnetic wave propagation in the region. This independent manipulation of the phase allows for a wide range of applications of chiral metasurfaces; a lens achieves monofocal distance by changing the phase.

### 2.2. Multifocal Lens

A monofocal lens metasurface can be obtained by arraying the phases calculated according to the above theory. However, this theoretical approach only applies to the focusing of a single focal point, and there are limitations in utilizing the method to achieve the focusing of multiple focal points. Holography is able to record and reconstruct all the information of the wavefront of a specific target or object, which is widely used in two-dimensional and three-dimensional images, large-capacity data storage, etc. It is a kind of image display technology with development prospects. Based on the principle of holographic image display, the multifocal phase change is derived as follows: the field distribution on the metasurface is a superposition of spherical waves from the target field, and in order to realize the target field, it is necessary to discretize the target field into multiple point sources, and the Green’s function *G*(*Rn*) describes the propagation of the electromagnetic field of each virtual point source. Assuming that n point sources are generated at location $F_{n}$, the superimposed electric field E at the metasurface is expressed as follows:

(5) $$E_{Fcous}\left(x_{i},y_{i},0\right)=\sum_{n=1}^{N}\left[W_{Fn}·G(R_{n})\right]$$

(6) $$E_{Fcous}\left(x_{i},y_{i},0\right)=\sum_{n=1}^{N}\left[W_{Fn}(x_{i},y_{i},0)·exp(jk_{0}R_{n})\right]$$

(7) $${\;W}_{Fn}\left(x_{i},y_{i},0\right)=\frac{A_{n}}{R_{n}}\;$$

(8) $$R_{n}=\left|F_{n}-ri\right|$$

where $\left(x_{i},y_{i},0\right)$ denotes the coordinates of the *i*-th chiral metasurface to the center, and $W_{Fn}$ denotes the intensity weighting coefficient of the *n*-th focal point. $A_{n}$ is a constant, denoting the intensity of the *n*-th point source. $R_{n}$ denotes the distance from the *n*-th point source to the *i*-th chiral metasurface cell, and $k_{0}$ denotes the wave number. Fn  denotes the distance from the *n*-th point source to the origin, $r_{i}\;$ denotes the distance from the *i*-th cell to the origin, and *N* is the total number of point sources.

The reference wave is a positively incident plane wave that arrives on the chiral metasurface with a constant electric field strength and phase. Assuming this constant phase to be $\varphi_{d}$, the chiral metasurface can be phase compensated to achieve the desired field distribution:

(9) $$\varphi \left(x_{i},y_{i},0\right)=\varphi_{d}-angleE_{Fcous}(x_{i},y_{i,}0)$$

According to the theoretical analysis, the number and position of the focal points can be designed as needed. Here, taking two focal points as an example, we chose to make the weighting coefficients equal in order to realize a uniform light intensity distribution. The spatial cumulative phase of the electromagnetic wave from the *i*-th cell to the chiral metasurface focal point can be calculated. The metasurface realizes a multifocal lens via the change of phase.

(10) $$\Phi \left(x_{i},y_{i},0\right)=\varphi_{d}-\varphi_{Fcous}\left(x_{i},y_{i},0\right)=\varphi_{d}-angle\sum_{n=1}^{2}\left[\frac{W_{Fn}·exp(jk_{0}W_{Fn}·exp(jk_{0}\left|F_{n}-r_{i}\right|))}{\left(jk_{0}\left|F_{n}-r_{i}\right|\right)}\right]$$

## 3. Model

The simulation software constructs the H model by scanning four structural parameters to generate the desired dataset. These parameters include the rotation angle on both sides of the rectangular column H (ranging from −20° to 50°), the length H1 (ranging from 30 µm to 70 µm), the length H2 (ranging from 30 µm to 90 µm), and the width w2 (ranging from 5 µm to 15 µm), resulting in a total of 840 datasets. The length H1 of the H-structure is transformed from 30 µm to 70 µm in steps set to 10 µm. With an assumed accuracy of 0.001, the CD values for the H structure at each length, ranging from 30 µm to 70 µm, can be calculated through 40,000 iterations. In this study, four structural parameters were changed, and the amount of computation required to simulate all these changed shapes and calculate the CD values would be very large. To calculate the CD values faster and more accurately, a neural network model is incorporated in this paper to identify, precisely, the best CD values for these scanned parameters and to design a chiral metasurface.

**Figure 1:**
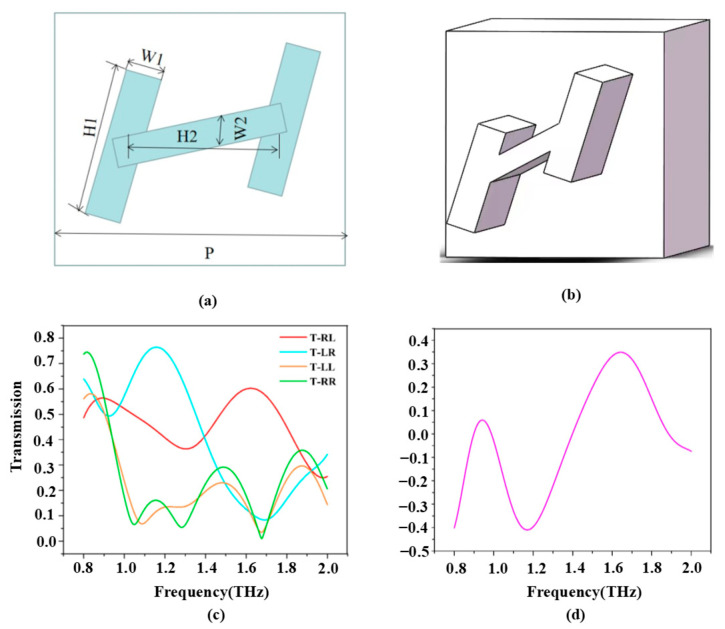
(**a**,**b**) Schematic diagram of the structure of the base atom. (**c**) Simulation of transmission coefficients for circular polarization. (**d**) Transmission CD of the base atom.

By manipulating the size and rotation angle of each rectangular column, the CD of the chiral metasurface can be adjusted, allowing for the desired designs. In this work, the asymmetric all-silicon H-type chiral metasurface was designed based on a deep learning-assisted design method. The structures we choose are generally H-shaped because they can easily by fabricated via top-down methods and are variable in both length and angle. This variable geometry is complex enough to cover a wide range of optical response spectra for both polarizations. The specific design parameters are as follows: the period P is set to 150 µm, the lengths of the rectangular columns H1 and H2 are determined as 69.745 µm and 74.743 µm, respectively. Furthermore, the widths of the rectangular columns, denoted as w1 and w2, are selected as 20 µm and 13.702 µm, respectively. Lastly, the entire H-shaped structure is rotated by 27°. Detailed visual representations of the designed chiral metasurface can be observed in Figure 1a,b.

The time-domain solver within the simulation software is employed to calculate the transmission coefficients of H-type structures when subjected to different polarized light irradiation. The chiral metasurface can realize the transmission-type polarization transformation. As shown in Figure 1c, when *LCP* is irradiated to the chiral metasurface, the transmission is colored in red (*LCP* incident *RCP* out) and orange (*LCP* incident *LCP* out), which shows that when *LCP* is incident to the chiral metasurface at 1.169 THz, there is a small amount of *RCP* in the outgoing wave. When the *RCP* is incident on the chiral metasurface, the transmission is colored in blue (*RCP* incident *LCP* out) and green (*RCP* incident *RCP* out), which shows that the *RCP* produces almost only *LCP* when it is incident on the chiral metasurface at 1.169 THz [44,45,46]. This also explains the subsequent discovery that only *RCP* incident produces focus. As shown in Figure 1d, in the design of the metasurface, the x and y directions are set as periodic boundaries, and the z direction is set as an open boundary. The study simulates the transmission coefficient of a single metasurface under different circularly polarized light and then calculates the CD using the following equation. At an operating frequency of 1.169 terahertz, light incident perpendicular to the metasurface produces a CD value of 0.41 for an all-silicon H-type structure.

(11) $$T_{CD}=T_{R}-T_{L}=\left({\left|t_{RR}\right|}^{2}+{\left|t_{LR}\right|}^{2}\right)-({\left|t_{LL}\right|}^{2}+{\left|t_{RL}\right|}^{2})$$

## 4. Network

The conditional generative adversarial network (CGAN) primarily comprises a generator (*G*) and a discriminator (*D*). The optimization objective of CGAN is as follows:

(12) $$\underset{G}{\min}\underset{D}{\max}V\left(D,G\right)=E_{X\sim P_{data}(x)}\left[logD(x)\right]+E_{Z\sim P_{Z}\left(Z\right)}\left[log(1-D(G\left(z|y\right)))\right]$$

**Figure 2:**
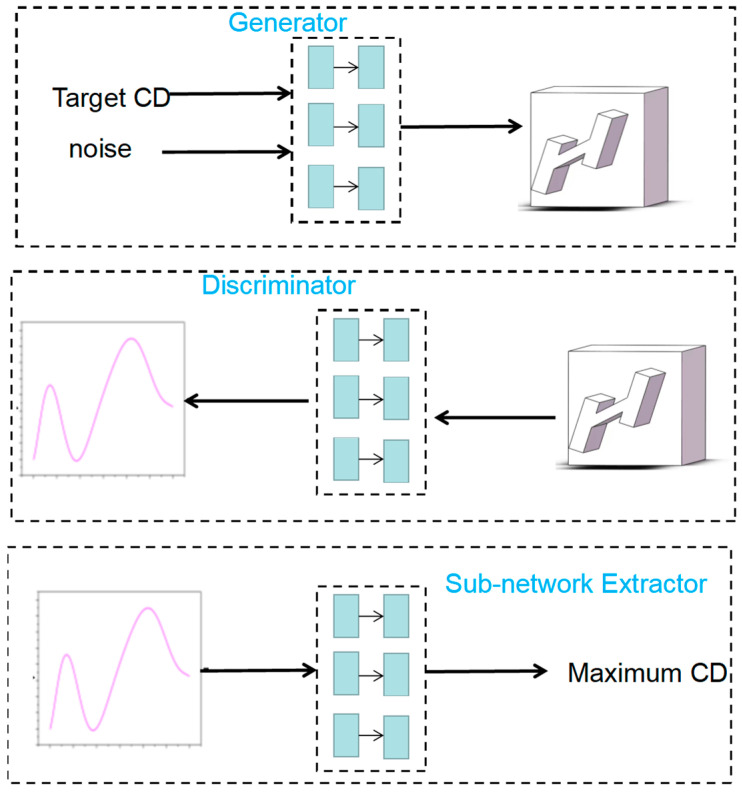
Schematic diagram of PCGN. It consists of three main parts: generator, discriminator, and sub-network extractor. The input to the generator in this paper consists of a maximum CD and random noise. The purpose of noise is that multiple sets of different structural parameters can be output to meet the design requirements.

When utilizing conditional generative adversarial networks (CGAN) for inverse design, some difficulties are encountered because the discriminator tends to have large errors in acquiring the structural parameters. This greatly increases the complexity of the training process. In order to solve this problem during the forward training process, this paper proposes a novel network structure by introducing a sub-network after the discriminator, as shown in Figure 2. The whole network consists of three parts: generator, discriminator, and sub-network extractor. The inputs of the generator are the target CD and a set of random noises, and the outputs are the structural parameters that can reach the target CD. In addition, the discriminator has as input the structural parameters, and, as output, the frequency-CD curve. A well-trained discriminator and sub-network extractor can obtain the CD and its maximum value for a given structure and provide guidance for the training of the generator. In addition, the trained generator can reverse-engineer the desired structural parameters by inputting the target CD and random noise and generate multiple sets of structural parameters that satisfy the target based on the variation of the random noise.

**Figure 3:**
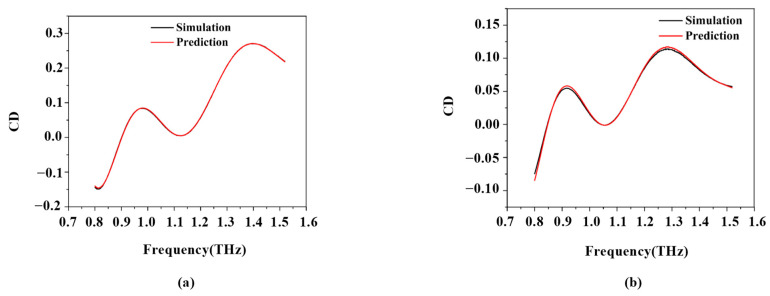
(**a**) Fit of training data. (**b**) Fit of test data.

As shown in Table 1, this network is the discriminator. Inputting the H-structure parameters into the eight fully connected layers gives the values of all CDs. In this network structure, 600 sets of data are used for training, and 240 sets of data are used for testing. The number of training rounds is 45,000, and the learning rate is 0.001. When this part of the model training is finished, a set of data trained with the network model is randomly selected and compared with the simulated data. The comparison results are shown in Figure 3a,b. The analysis shows that the data trained by the network model and the simulated data are basically the same. This network has an MAE of 0.0017, and the network is great.

As shown in Table 2, this network is a sub-network extractor, taking as input all the CD values obtained from the discriminator network. The maximum CD can be obtained by five fully connected layers. The learning rate designed in this network structure is 0.001, and its MAE is 0.0031 in the network structure at the end of training, which is a great network effect.

**Figure 4:**
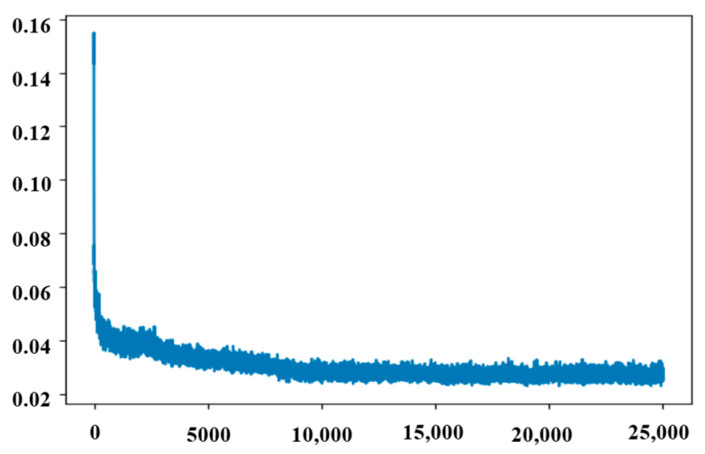
Errors in inverse networks.

As shown in Table 3, this network is the generator. The activation function used in the network was ELU. The chiral metasurface structure parameters were obtained through three fully connected layers using the maximum CD predicted by the sub-network as input. As shown in Figure 4, the MAE of this network is 0.0223 at the end of training.

**Figure 5:**
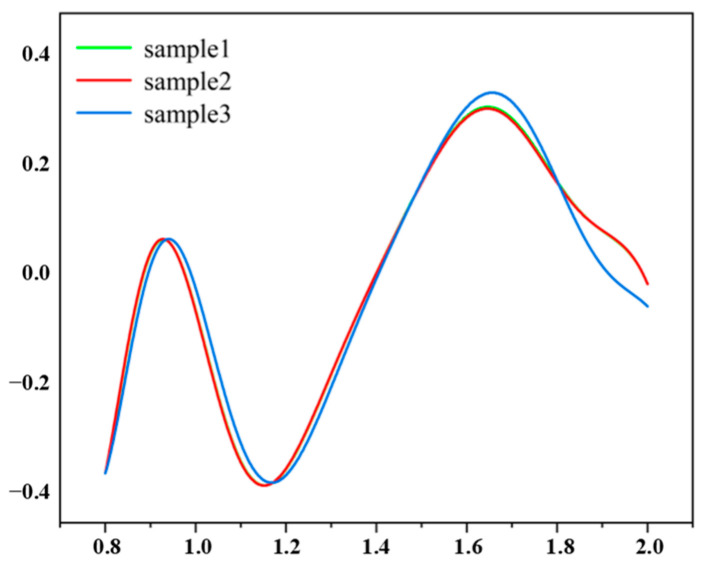
The CD spectra of three chiral metasurface structures.

After the generator has been trained, the trained generator can also reverse design the desired structural parameters by inputting the target CD and random noise and generating multiple sets of structural parameters that satisfy the target based on the variation of the random noise. In order to verify the generator training effect, here, we input the target CD value of 0.41 and randomly generate noise in this network to reverse design multiple sets of structural parameters that satisfy the target value, as shown in Table 4. The CD values obtained for the three sets of structural parameters are shown in Figure 5. It can be seen from the figure that the three structures have the same CD values obtained at a frequency of 1.169 THz. From these three sets of data, it can be seen that the generator network is well-trained.

To alleviate the highly nonlinear problem of gradient disappearance and inverse design, ELU is used as the activation function in this paper. The mean value of the ELU function output is centered at 0. When *x* takes a negative value, the ELU is saturated and thus has some robustness with respect to noise. The ELU function is shown below:

(13) $$f\left(x\right)=\left\{\begin{array}{c} x\;,x\geq 0\\ a\left(e^{x}-1\right),x<0 \end{array}\right.$$

Preprocessing plays a very important role in deep learning. To make it easier to recover the predicted data when using the network, the input parameters are preprocessed using normalization methods. This can be expressed as follows:

(14) $$x=\frac{x_{0}-minx_{0}}{maxx_{0}-minx_{0}}\;$$

When the training is over, the values output in the network are as follows:

(15) $$x_{0}=x\left(maxx_{0}-minx_{0}\right)+minx_{0}$$

Optimizers, as an important part of deep learning, often produce unanticipated effects when networks are trained on large amounts of data. Sometimes an incorrect choice of optimizer may result in the network model not being trained properly. It is necessary to choose a suitable optimizer during our training process. Adaptive moment estimation calculus (Adam) is chosen in this study; this algorithm corrects for bias and is adaptive to the learning rate. The Nadam computation (Nadam) can perform gradient descent optimization and is an extension of the adaptive estimator optimization algorithm. These two optimizers are now widely used in deep learning.

In deep learning, the error between a single training sample and the true value is known as the loss function. The loss function is not unique for various networks. In practical applications, the loss function is selected according to the desired design goal to achieve the best network performance. The main loss function used in this paper is mean absolute error (MAE). MAE is also known as L1 loss, which is calculated as the absolute value of the average error between the true value and the predicted value, and the purpose of modeling the average error is to make sure that the errors do not cancel each other out in the process of calculation.

## 5. Result

**Figure 6:**
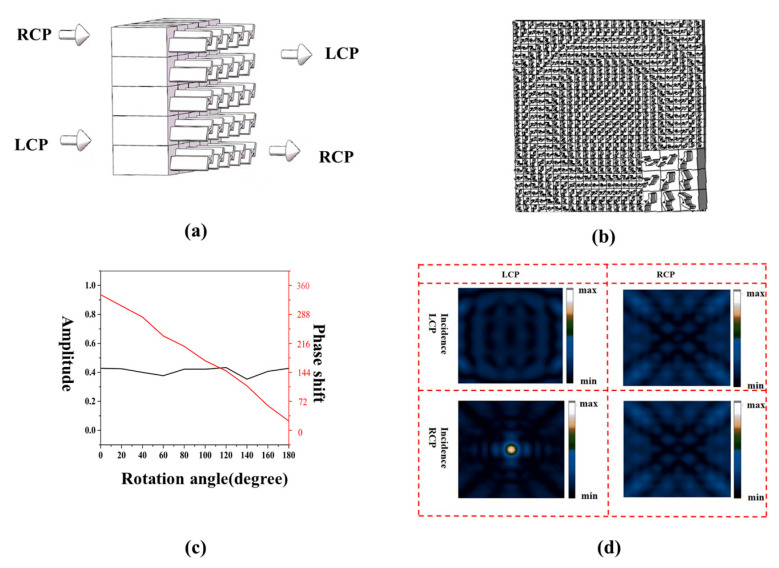
(**a**) Schematic diagram of the structural parameters of the chiral metasurface. (**b**) Schematic diagram of an array consisting of 25 × 25 primitives drawn with simulation software. The inset is a partially enlarged schematic diagram. (**c**) Schematic diagram of the simulation results under RCP incidence. The red curve is the phase of the output LCP wave, and the black curve is the amplitude of the output LCP wave. (**d**) Output LCP/RCP simulated intensity distribution when the incident light is RCP/LCP.

At a frequency of 1.169 terahertz, the analysis was performed by rotating the basic atom by a specific angle. As shown in Figure 6c, as the substrate atoms are rotated from 0° to 180°, the transmission amplitude remains essentially constant, while the cross-circular polarization phase changes from 0° to 360°. The red curve in Figure 6 shows a strong linear relationship between these values. This shows that the simulation results validate the accuracy of the PB phase theory. As shown in Figure 6a,b, the chiral metasurface array consists of 25 × 25 base atoms. When LCP is incident to the chiral metasurface array, the output electric field intensity RCP is shown in Figure 6d. When RCP is incident to the chiral metasurface array, the output electric field intensity LCP is shown in Figure 6d. It can be seen that focusing can be produced when RCP is incident to the chiral metasurface array.

**Figure 7:**
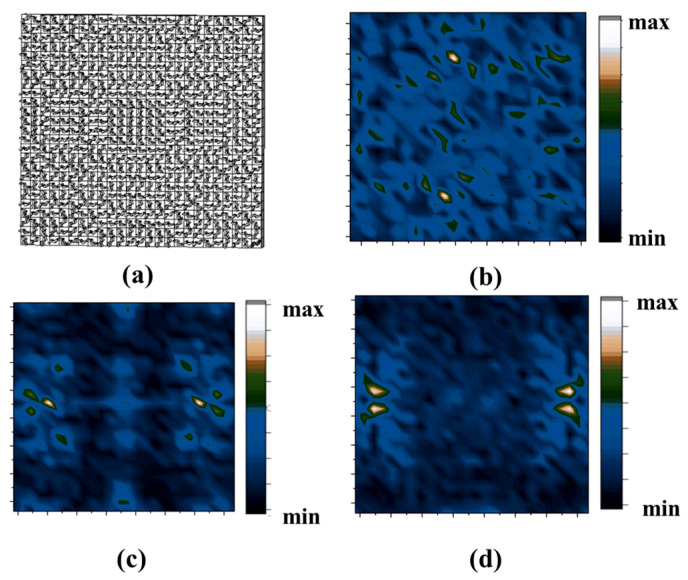
(**a**) Schematic drawing of an array of 25 × 25 primitives using simulation software. (**b**,**c**) The controllability of the metasurface focusing position, changing the focal point coordinates. (**d**) The focusing of four foci is achieved on the metasurface.

As shown in Figure 7a, the chiral metasurface is composed of a 25 × 25 matrix according to the phase variation of Equation (9). Holographic theory calculates how the phases on the metasurface change. The incident plane wave can converge to a specified point in an arbitrary way, including the number of foci, position, and intensity distribution, with excellent maneuverability. By carefully designing the phase distribution on the chiral metasurface, it is possible to realize the focusing of the lens at different positions as well as to realize the focusing of multiple foci, as shown in Figure 7b–d. It is shown in the results that the designed metasurfaces have good multifocal focusing characteristics. The H-structure of the metasurface lens is a 25 × 25 matrix, and if there are more units, the phase information contained on the metasurface will be richer and the focusing effect will be better.

## 6. Conclusions

In conclusion, an all-silicon chiral metasurface is designed using deep learning techniques. The proposed PCGN network, based on the CGAN model, is capable of inverse design for different chiral metasurface structures via its training with diverse structural data. The network accurately predicts the CD, achieving a CD value of 0.41 at a frequency of 1.169 THz. By manipulating the asymmetric transmission characteristics of RCP and LCP light, a polarization conversion efficiency of 95% is achieved at a CD value of 0.41. At this frequency, using the Pancharatnam–Berry (PB) principle, phase modulation can be performed with a right circularly polarized wave against a chiral metasurface array, thereby realizing a monofocal lens. Based on the principle of holographic image display, the variation of the multifocal phase is analyzed, and the focus position and the number of generated point sources can be calculated, thus realizing a multifocal lens. The proposed multifocal lens metasurface shows great potential for applications in antennae and imaging systems. Compared with conventional methods, the on-demand design method proposed in this study reduces the waste of computational resources and eliminates the need to repeat simulations to fine-tune the structural parameters.

## Figures and Tables

**Table 1:** Structure of discriminator.

Layers	Dimensions	Normalization	Activation Function
Input	256 × 1	\	\
Hidden Layer 1	256 × 1	Batch Norm	ELU
Hidden Layer 2	256 × 1	Batch Norm	ELU
Hidden Layer 3	256 × 1	Batch Norm	ELU
Hidden Layer 4	256 × 1	Batch Norm	ELU
Hidden Layer 5	256 × 1	Batch Norm	ELU
Hidden Layer 6	256 × 1	Batch Norm	ELU
Output	256 × 1	Batch Norm	ELU

**Table 2:** Structure of sub-network extractor.

Layers	Dimensions	Normalization	Activation Function
Input	256 × 1	\	\
Hidden Layer 1	256 × 1	Batch Norm	ELU
Hidden Layer 2	256 × 1	Batch Norm	ELU
Hidden Layer 3	256 × 1	Batch Norm	ELU
Output	256 × 1	Batch Norm	ELU

**Table 3:** Structure of generator.

Layers	Dimensions	Normalization	Activation Function
Input	256 × 1	\	\
Hidden Layer 1	256 × 1	Batch Norm	ELU
Output	256 × 1	Batch Norm	ELU

**Table 4:** Parameters of the three chiral metasurface structures.

	H1	H2	W2	AL
Sample 1	69.9546576	87.988344	14.931272	49.9994120
Sample 2	69.971556	88.5941004	14.9732214	50.0000209
Sample 3	69.9740052	77.532534	13.7295526	49.9994976

## Data Availability

Research data presented in this study are available on request from the corresponding author.

## References

[1] Kim H.S., Baik S.Y., Lee J.W., Kim J., Ahn Y.H. (2021). Nondestructive Tomographic Imaging of Rust with Rapid THz Time-Domain Spectroscopy. Appl. Sci..

[2] Zhang M., Xie X., Zhang D., Chen R., Xu Y., Wang J., Liu J., Xu X. (2022). Nondestructive identification of wood species by terahertz spectrum. Microw. Opt. Technol. Lett..

[3] Hernandez-Cardoso G.G., Amador-Medina L.F., Gutierrez-Torres G., Reyes-Reyes E.S., Benavides Martinez C.A., Cardona Espinoza C., Arce Cruz J., Salas-Gutierrez I., Murillo-Ortiz B.O., Castro-Camus E. (2022). Terahertz imaging demonstrates its diagnostic potential and reveals a relationship between cutaneous dehydration and neuropathy for diabetic foot syndrome patients. Sci. Rep..

[4] Yin S., Ji W., Xiao D., Li Y., Li K., Yin Z., Jiang S., Shao L., Luo D., Liu Y.J. (2019). Intrinsically or extrinsically reconfigurable chirality in plasmonic chiral metasurfaces. Opt. Commun..

[5] Tanaka K., Arslan D., Fasold S., Steinert M., Sautter J., Falkner M., Pertsch T., Decker M., Staude I. (2020). Chiral Bilayer All-Dielectric Metasurfaces. ACS Nano.

[6] Yin S., Chen Y., Quan B., Liu S., Huang W., Liu M., Zhang W., Han J. (2023). Coupling-enabled chirality in terahertz metasurfaces. Nanophotonics.

[7] Pan R., Liu Z., Zhu W., Du S., Gu C., Li J. (2021). Asymmetrical Chirality in 3D Bended Metasurface. Adv. Funct. Mater..

[8] Li Q., Fan H., Bai Y., Li Y., Ikram M., Wang Y., Huo Y., Zhang Z. (2022). Deep learning for circular dichroism of nanohole arrays. New J. Phys..

[9] Tao Z., You J., Zhang J., Zheng X., Liu H., Jiang T. (2020). Optical circular dichroism engineering in chiral metamaterials utilizing a deep learning network. Opt. Lett..

[10] Fan Z., Govorov A.O. (2011). Helical Metal Nanoparticle Assemblies with Defects: Plasmonic Chirality and Circular Dichroism. J. Phys. Chem. C.

[11] Khaliq H.S., Kim I., Zahid A., Kim J., Lee T., Badloe T., Kim Y., Zubair M., Riaz K., Mehmood M.Q. (2021). Giant chiro-optical responses in multipolar-resonances-based single-layer dielectric metasurfaces. Photonics Res..

[12] Yue Z., Zheng C., Li J., Li J., Liu J., Wang G., Chen M., Xu H., Tan Q., Zhang H. (2021). A dual band spin-selective transmission metasurface and its wavefront manipulation. Nanoscale.

[13] Bokhari S.H.A., Cheema H.M. (2021). A Bilayered, Broadband, Angularly Robust Chiral Metasurface for Asymmetric Transmission. IEEE Antennas Wirel. Propag. Lett..

[14] Dicandia F.A., Genovesi S. (2023). Design of a Transmission-Type Polarization-Insensitive and Angularly Stable Polarization Rotator by Using Characteristic Modes Theory. IEEE Trans. Antennas Propag..

[15] Dicandia F.A., Genovesi S. (2022). Linear-to-Circular Polarization Transmission Converter Exploiting Meandered Metallic Slots. IEEE Antennas Wirel. Propag. Lett..

[16] Zhou J., Chowdhury D.R., Zhao R., Azad A.K., Chen H.-T., Soukoulis C.M., Taylor A.J., O’Hara J.F. (2012). Terahertz chiral metamaterials with giant and dynamically tunable optical activity. Phys. Rev. B.

[17] Burch J., Wen D., Chen X., Di Falco A. (2017). Conformable Holographic Metasurfaces. Sci. Rep..

[18] Wan W., Gao J., Yang X. (2017). Metasurface Holograms for Holographic Imaging. Adv. Opt. Mater..

[19] Song G.Y., Huang B., Dong H.Y., Cheng Q., Cui T.J. (2016). Broadband Focusing Acoustic Lens Based on Fractal Metamaterials. Sci. Rep..

[20] Wang Q., Zhang X., Xu Y., Tian Z., Gu J., Yue W., Zhang S., Han J., Zhang W. (2015). A Broadband Metasurface-Based Terahertz Flat-Lens Array. Adv. Opt. Mater..

[21] Chen X., Zhang Y., Huang L., Zhang S. (2014). Ultrathin Metasurface Laser Beam Shaper. Adv. Opt. Mater..

[22] He J., Ye J., Wang X., Kan Q., Zhang Y. (2016). A broadband terahertz ultrathin multi-focus lens. Sci. Rep..

[23] Gu B., Yang G., Dong B. (1986). General theory for performing an optical transform. Appl. Opt..

[24] Liu F., Zhang W., Sun Y., Liu J., Miao J., He F., Wu X. (2020). Secure Deep Learning for Intelligent Terahertz Metamaterial Identification. Sensors.

[25] Ma W., Liu Z., Kudyshev Z.A., Boltasseva A., Cai W., Liu Y. (2020). Deep learning for the design of photonic structures. Nat. Photonics.

[26] Ma W., Cheng F., Liu Y. (2018). Deep-Learning-Enabled On-Demand Design of Chiral Metamaterials. ACS Nano.

[27] Girshick R., Kokkinos I., Laptev I., Malik J., Papandreou G., Vedaldi A., Wang X., Yan S., Yuille A. (2017). Editorial-Deep Learning for Computer Vision. Comput. Vis. Image Underst..

[28] Voulodimos A., Doulamis N., Doulamis A., Protopapadakis E. (2018). Deep Learning for Computer Vision: A Brief Review. Comput. Intell. Neurosci..

[29] Jayalaxmi P.L.S., Saha R., Kumar G., Kim T.-H. (2022). Machine and deep learning amalgamation for feature extraction in Industrial Internet-of-Things. Comput. Electr. Eng..

[30] Liang H., Sun X., Sun Y., Gao Y. (2017). Text feature extraction based on deep learning: A review. EURASIP J. Wirel. Commun. Netw..

[31] Sorin V., Barash Y., Konen E., Klang E. (2020). Deep Learning for Natural Language Processing in Radiology-Fundamentals and a Systematic Review. J. Am. Coll. Radiol..

[32] Otter D.W., Medina J.R., Kalita J.K. (2021). A Survey of the Usages of Deep Learning for Natural Language Processing. IEEE Trans. Neural. Netw. Learn. Syst..

[33] Teng Y., Li C., Li S., Xiao Y., Jiang L. (2023). Efficient design method for terahertz broadband metasurface patterns via deep learning. Opt. Laser Technol..

[34] Wu B., Wang G., Liu K., Hu G., Xu H.-X. (2022). Equivalent-circuit-intervened deep learning metasurface. Mater. Des..

[35] An S., Zheng B., Julian M., Williams C., Tang H., Gu T., Zhang H., Kim H.J., Hu J. (2022). Deep neural network enabled active metasurface embedded design. Nanophotonics.

[36] An S., Fowler C., Zheng B., Shalaginov M.Y., Tang H., Li H., Zhou L., Ding J., Agarwal A.M., Rivero-Baleine C. (2019). A Deep Learning Approach for Objective-Driven All-Dielectric Metasurface Design. ACS Photonics.

[37] Ghorbani F., Shabanpour J., Beyraghi S., Soleimani H., Oraizi H., Soleimani M. (2021). A deep learning approach for inverse design of the metasurface for dual-polarized waves. Appl. Phys. A.

[38] Zheng B., Zhang H. Deep Learning Based Multi-layer Metallic Metasurface Design. Proceedings of the 2020 IEEE International Symposium on Antennas and Propagation and North American Radio Science Meeting.

[39] Naseri P., Hum S.V. (2021). A Generative Machine Learning-Based Approach for Inverse Design of Multilayer Metasurfaces. IEEE Trans. Antennas Propag..

[40] Chen Y., Zhu J., Xie Y., Feng N., Liu Q.H. (2019). Smart inverse design of graphene-based photonic metamaterials by an adaptive artificial neural network. Nanoscale.

[41] Hasman E., Kleiner V., Biener G., Niv A. (2003). Polarization dependent focusing lens by use of quantized Pancharatnam–Berry phase diffractive optics. Appl. Phys. Lett..

[42] Zhang Y., Pu M., Jin J., Lu X., Guo Y., Cai J., Zhang F., Ha Y., He Q., Xu M. (2022). Crosstalk-free achromatic full Stokes imaging polarimetry metasurface enabled by polarization-dependent phase optimization. Opto-Electron. Adv..

[43] Huang Y., Xiao T., Chen S., Xie Z., Zheng J., Zhu J., Su Y., Chen W., Liu K., Tang M. (2023). All-optical controlled-NOT logic gate achieving directional asymmetric transmission based on metasurface doublet. Opto-Electron. Adv..

[44] Li J., Yue Z., Li J., Zheng C., Liu J., Yang F., Li H., Zhang Y., Zhang Y., Yao J. (2022). Wavefront-controllable all-silicon terahertz meta-polarizer. Sci. China Mater..

[45] Huang Y., Xiao T., Xie Z., Zheng J., Su Y., Chen W., Liu K., Tang M., Müller-Buschbaum P., Li L. (2020). Single-Layered Reflective Metasurface Achieving Simultaneous Spin-Selective Perfect Absorption and Efficient Wavefront Manipulation. Adv. Opt. Mater..

[46] Zhang F., Pu M., Li X., Gao P., Ma X., Luo J., Yu H., Luo X. (2017). All-Dielectric Metasurfaces for Simultaneous Giant Circular Asymmetric Transmission and Wavefront Shaping Based on Asymmetric Photonic Spin–Orbit Interactions. Adv. Funct. Mater..

